# Evaluation of an inter-professional workshop to develop a psychosocial assessment and child-centred communication training programme for paediatricians in training

**DOI:** 10.1186/1472-6920-4-25

**Published:** 2004-11-21

**Authors:** Debra Nestel, Sharon Taylor, Quentin Spender

**Affiliations:** 1 Department of Psychological Medicine, Imperial College London, United Kingdom; 2 Imperial College, St Mary's Higher Training Scheme, United Kingdom; 3 Chichester & St George's Hospital Medical School, United Kingdom

## Abstract

**Background:**

The quality of psychosocial assessment of children in consultations varies widely. One reason for this difference is the variability in effective mental health and communication training at undergraduate and post-qualification levels. In recognition of this problem, the Royal College of Paediatrics and Child Health in the United Kingdom have developed the Child in Mind Project that aims to meet this deficit in medical training. This paper describes the evaluation of a workshop that explored the experiences and expectations of health care professionals in the development of a training programme for doctors.

**Methods:**

The one-day inter-professional workshop was attended by 63 participants who were invited to complete evaluation forms before and immediately after the workshop.

**Results:**

The results showed that the workshop was partially successful in providing an opportunity for an inter-professional group to exchange ideas and influence the development of a significant project. Exploring the content and process of the proposed training programme and the opportunity for participants to share experiences of effective practice were valued. Participants identified that the current culture within many health care settings would be an obstacle to successful implementation of a training programme. Working within existing training structures will be essential. Areas for improvement in the workshop included clearer statement of goals at the outset and a more suitable environment for the numbers of participants.

**Conclusions:**

The participants made a valuable contribution to the development of the training programme identifying specific challenges. Inter-professional collaborations are likely to result in more deliverable and relevant training programmes. Continued consultation with potential users of the programme – both trainers and trainees will be essential.

## Background

Childhood mental health disorders are common. A Department of Health survey of 5 to 15 year-olds in England and Wales, 5% had conduct disorder, 4% had emotional disorders and 1% rated as hyperactive [1]. However, during consultations the psychosocial assessment of children is sometimes compromised. The reasons are varied and most often reflect deficits in relevant knowledge, attitudes and skills of health care professionals. Children are often not placed at the centre of the consultation [2-7]. Further, health care professionals have been found to lack knowledge in pain management of children [8,9] and are poor at giving information to children and adolescents with cancer [10] which may lead to poor adjustment to illness and emotional problems. The consequences for children and their families can be profound.

Knowledge of psychosocial and mental health problems is only part of the patient assessment process. The ability to communicate effectively with the patient is pivotal for accurate assessment [11-13]. A unique feature of the paediatric interview is its triadic structure. That is, consultations often involve a child, their parent and a doctor. However, research in paediatric interviewing usually deals with one dyad (parent-doctor), sometimes two dyads (parent-doctor and child-doctor) or even three dyads (parent-doctor and child-doctor and parent-child) rather than a triad of parent-doctor-child [14].

New graduates in the United Kingdom are expected to be able to "communicate clearly, sensitively and effectively with patients and their relatives" [15]. Teaching and learning about medical interviewing is now part of mainstream undergraduate medical education [16]. Acquisition of medical interviewing skills should not stop at graduation. Continuing professional development supports the maintenance and extension of patient-centred interviewing skills. Although summative assessments of interviewing skills are well established in some Royal Colleges [17], they are being incorporated in others [18]. Training programmes to support doctors in these summative assessments are developing simultaneously. In the United Kingdom there is scant evidence that medical interviewing programmes at any level consider the specialised skills required for triadic interviewing associated with paediatric consultations. However, studies from Europe and North America report educational interventions that incorporate triadic interviewing [19-21] while others focus on the dyad (e.g. doctor-parent; doctor-child; doctor-adolescent) [22,23].

In order to address these two issues, the Child in Mind Project at the Royal College of Paediatrics and Child Health *(RCPCH) *aims to develop psychosocial awareness and interviewing skills of paediatricians (in training). This will improve the assessment and management of psychosocial issues that effect children. To achieve this goal, a modular training programme in child and adolescent mental health is proposed. The programme will be piloted with senior house officers (SHOs) on paediatric rotations. Building additional modules into existing SHO training programmes is problematic since clinical and other commitments already consume a shrinking working week. Therefore, a key consideration in developing the training modules is to work within existing SHO training by maximising both planned and opportunistic teaching and learning in psychosocial care and interviewing skills appropriate for working with children, adolescents and their families.

At an early stage in the project, the team thought it appropriate to elicit ideas and experiences of interested professionals as well as recruit individuals to help with developing the project. An open invitation to attend a one-day workshop held at the RCPCH was advertised in relevant professional newsletters for paediatricians, child psychiatrists, and child psychologists and by word of mouth in other disciplines. The invitation stated that the workshop would elicit the views of an inter-professional group interested in developing a training programme to improve psychosocial assessment and child-centred communication skills of doctors. Participants were chosen from twice the number of applicants to represent a balance of disciplines and geographical spread. Selection within these criteria was made on the order of receipt of applications. Numbers were limited by the capacity of the College facilities.

This paper describes the evaluation of the inter-professional workshop in the development of the training programme.

### Description of workshop

The opening plenary session introduced the Child in Mind Project together with the aims of the workshop (Table 1). These aims reflected the preliminary work the Project team had undertaken as well as their areas of content expertise. Four parallel group sessions then focused on core content and process issues for paediatric trainees: communication and interviewing skills, management of children and adolescents with recurrent aches and pains and intentional overdose. Topic experts were invited by the RCPCH to facilitate each group. The structure and methods used in the groups varied. Participants were allocated to specific sessions so that there were approximately equal numbers representing all disciplines present in each group.

**Table 1:** Participants' ratings of the helpfulness of the sessions in meeting the aims of the workshop (n = 28)

	**Not at all helpful**	**Partially helpful**	**Very helpful**
**Plenary Session 1**		14	11
Introduction, background, aims			
**Group Sessions – Content**			
1. How can we teach communication & interview skills?		3	
2. How can we teach communication & interview skills?	1	3	3
3. How can we teach the management of recurrent aches and pains?	2	7	
4. How can we teach the management of intentional overdose?		4	2
**Plenary Session 2**	2	17	6
Feedback from morning sessions, planning for afternoon			
**Group Sessions – Process**			
1. How can we get trainees to role-play?		4	2
2. How can we combine traditional teaching and learning methods with new technology?	2	1	3
3. How can we integrate child mental health into existing training programmes?		7	3
4. How can we assess paediatric trainees in child mental health?	1	5	
Plenary Session 3		11	6
Feedback from afternoon sessions, conclusions, action			

Immediately after lunch, a plenary session was held in which the key issues from the morning sessions were shared with the entire group. The four parallel sessions that followed focused on core process issues in training: promoting role-play, introducing technology, integrating new with existing programmes, and assessment. The final plenary session provided an opportunity for groups to share their experiences and then a wider discussion considered key issues from both morning and afternoon sessions. An action plan was devised based on this discussion and was shared with participants providing an opportunity for participants to continue to be involved in the project.

## Methods

**Figure 1:**
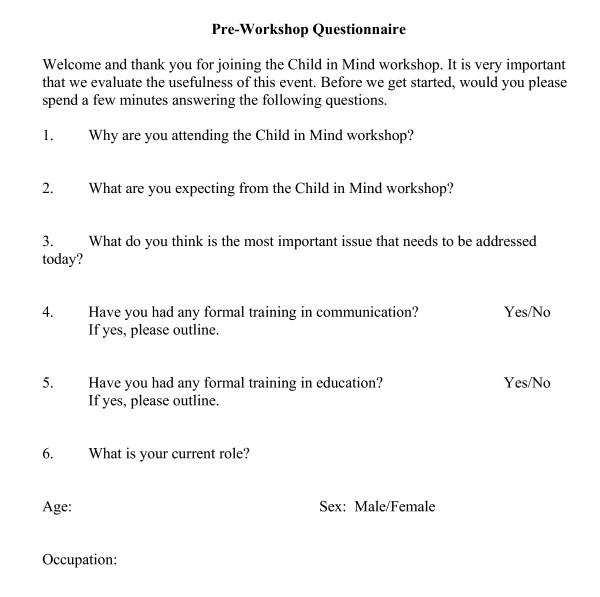
Pre-Workshop Evaluation Form

**Figure 2:**
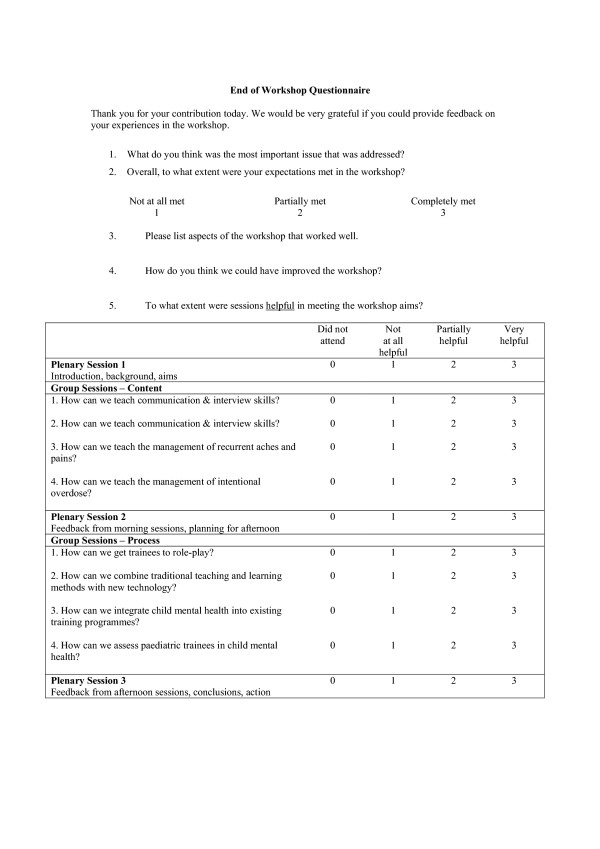
Post-Workshop Evaluation Form

All participants were invited to complete evaluation forms immediately before and after the workshop. The pre workshop form explored participants' reasons for attending, their expectations, their most important issue in relation to the workshop, their experiences in learning about communication and education, their current role, age and sex (Figure 1). The post workshop form was divided into two parts (Figure 2). The first part asked participants' about their experiences of the workshop, the most important issue that was covered, the degree to which their expectations were met, the aspects of the workshop that went well and those that could have been improved. The second part asked them to rate each session in relation to whether it was helpful in meeting the aims of the workshop. All responses were anonymous.

## Results

Sixty-three participants attended the workshop of whom 23 were clinical psychologists (36.5%), 18 paediatricians (28.6%), 9 psychiatrists (14.3%), 9 nurses (14.3%) and one representative from each of the following professions: social work, education, play therapy and occupational therapy (6.4%). Forty-one participants were female (65.1%) and 22 were male (34.9%). Approximately twenty participants (31.8%) left the workshop immediately prior to the closing plenary session. This was unexpected and apparently not triggered by anything more than a need to catch commuter and intercity trains. That is, the exodus seemed unrelated to the quality of the meeting. Twenty-two participants (34.9%) completed the pre workshop form and 28 completed the post workshop form (44.4%).

### Pre workshop evaluation

Of the 22 respondents completing the pre workshop form, 17 were female (77.3%) and 5 were male (22.7%) with an age range of 34 to 58 years and mean age of 45. Although the group were inter-professional, the respondents were predominantly medical with 10 paediatricians (45.5%), 5 child and adolescent psychiatrists (22.7%), 4 clinical psychologists (18.2%), one occupational therapist (4.5%) and one educator (4.5%).

Nineteen (86.4%) participants reported previous formal training in communication as part of undergraduate, post-graduate and continuing professional development. Training included theoretical and skills practice within and outside of paediatrics at fundamental (e.g. presentation skills, psychology training) and advanced levels (e.g. Balint groups, psychology training, bereavement). Eighteen participants (81.1%) reported at least some previous formal training in education.

#### Reasons for attending the workshop

Participants' reasons for attending the workshop were diverse and included a strong interest and or experience in the major themes of the workshop – assessment and management of psychosocial issues in paediatrics, paediatric interviewing and training.

To enhance the "voice of the child" in paediatrics. (4)

I have a long standing interest and involvement in the teaching of junior paediatricians the skills of communication, family therapy and management of behavioural and emotional issues. I have been trying to find ways of formalizing mental health training for paediatricians. (1)

Because I have a very real interest in improving the awareness and training of paediatricians in the psychosocial aspects of paediatrics. (7)

To participate in the development of paediatric psychological training. (17)

To help develop teaching of mental health issues in childhood. (18)

Having worked in the area of paediatric psychology for some years, I am particularly interested in developing the awareness of paediatricians re psychological issues. (19)

To contribute to the planning of teaching paediatricians how to tackle social and emotional issues. (20)

To better understand needs of paediatricians for training in psychological needs of children and families. (11)

Some participants wanted to develop existing local programmes.

To feedback ideas to our paediatric College/Clinical Tutor who did not get a place at the course. (12)

To try and improve our in-house teaching of psychological factors in paediatrics. (13)

One participant acknowledged a deficit in current training.

I realize we are generally very poor at integrating psychological aspects of child and family health into the busy acute training programme. (2)

#### Expectations of the workshop

The second question asked participants what they were expecting from the workshop. Various themes emerged and included the generation of ideas for the Child in Mind project generally and specifically in the development of training materials. Participants expected to be able to exchange ideas on what and how to change existing training and there was an expressed desire not only to influence these developments but to ensure they are deliverable.

To meet, share and hopefully influence colleagues. (17)

To participate in putting together relevant training modules and to have a voice in the future training of paediatricians. (19)

To ensure that programmes will be acceptable. To broaden ideas around what to include in communication programme. (5)

To offer my experience in direct work with children, adolescents and families. (9)

An understanding of where the project is so far – aims, methods, plans. A chance to contribute. (14)

A second theme related to expectations of inter-professional collaboration both in the development and delivery of the training module and the third and overlapping theme focussed on the opportunity for networking.

Decrease inter-professional tension and enhance collaboration. (4)

Participants' perceptions of the most important issue to be addressed in the workshop reflected their different expectations. Training issues were dominant and focused on both the content and process. Content issues included thinking about ways of raising the significance of the assessment and management of psychological problems in children and adolescents together with the need to identify the child and adolescent's perspective separately to their family's and health care professionals.

Developing a culture of respect for child and family. Accessing children's thoughts and feelings independently of their parents or other professionals. (4)

Process issues included identifying ways to maximise existing expertise, to use limited resources efficiently, to encourage participation from paediatric trainers and trainees and to consider assessment and evaluation as integral to the training programme.

### Post workshop evaluation

Twenty-eight (44.4%) participants completed the post workshop form. Demographic data was not collected. The "did not attend" option on the evaluation form is not included in Table 1 because participants did not use it. Instead, they indicated the parallel session that they attended by rating it. Ratings were consistent with the numbers of forms received. That is, 25 (89.2%) for plenary sessions 1 & 2 and the morning group sessions while 28 (100%) rated the afternoon group sessions and 17 (60.7%) rated plenary session 3.

Using a 3-point scale from not at all, partially to completely, participants rated the helpfulness of the sessions in meeting the objectives of the workshop (Table 1). The majority of participants rated the sessions as at least partially helpful. The qualitative data provided insight into participants' ratings.

#### Most important issues

Respondents were asked to identify the most important issues that they thought had been addressed in the workshop. Several participants wrote of the need to change the existing culture to one in which psychosocial assessment and communication skills are valued. There was also acceptance of diversity in workplaces and the training offered therein. To ensure that the new training programme is deliverable it must be sufficiently flexible to fit within these diverse settings and that it must be evaluated. The need for training both supervisors and trainers was considered requisite for implementing any programme.

Learning about the hospital paediatric culture and previous difficulties of teaching SHOs and getting the culture right. (20)

The importance of mental health teaching/learning for all doctors caring for children/families. (2)

Delivering training for trainers and that the child mental health programme needs to be integrated into existing paediatric training. (3)

Need to address appropriate training and supervision of SHOs and for consultants to be trained first themselves. (5)

The importance of introducing a general shift. The extreme inflexibility of the system as a whole. (9)

The realities of teaching busy SHOs who are preoccupied with passing exams. (14)

Changing culture of consultants to understand importance of training for mental health and communication skills. (24)

Some participants valued the opportunity to learn about existing effective practices while others gave consideration to who should teach, how and that whatever is taught must be relevant.

The importance of taking a full history and empowering SHOs to ask difficult questions, to reflect on their practice and to have supervision in order to understand what to do with the information they have gathered. (17)

Introduction of video review of consultations/interactions with children and parents to paediatrics. (22)

#### Meeting expectations

Participants used a 3-point scale from not at all, partially to completely to rate the degree to which they met their expectations. Eight participants completely met their expectations (28.6%) while twenty participants (71.4%) partially met their expectations.

#### What worked well

In response to being asked what worked well in the workshop, participants identified the opportunity to exchange ideas with colleagues with different levels of experience, who work in different settings and have different professional backgrounds.

The group sizes for sessions were valued since they were sufficiently small to enable several participants to express their views and large enough for diverse experiences to be shared. The plenary sessions were helpful in summarising group sessions and consolidating broad ranging issues.

The enthusiasm of delegates was thought to contribute to the success of the workshop together with the relaxed atmosphere and the genuine desire of participants and organisers to change existing practices.

#### Improvements to workshop

Most participants recorded at least one response to being asked how the workshop could be improved. The single most frequently cited issue related to the venue. Groups were too large for their rooms and for two groups, their presence in the same large room impeded discussion.

Other improvements included stronger facilitation in some groups to ensure all views were heard and that the discussion stayed focused. Providing delegates with basic information prior to the workshop on the aims, objectives and content of group sessions could have improved the quality of the discussions. Participants expressed a desire to attend the group sessions of their choice. One participant thought that the workshop was too rigidly organised between content and process and that this limited creativity in thinking about training. Two participants suggested including SHOs for whom the training will initially be delivered.

I felt unable to contribute much of my experience and knowledge with the tight preset agenda. I was particularly wanting to discuss raising awareness of child protection issues, and working with children in complex and or chaotic home situations. Also were there many current paediatric SHOs here today. If not, there should have been. If so, could they have contributed more? (17)

Sign up for preferred workshops on arrival – I don't remember what preferences I indicated but they certainly weren't the ones I was allocated. More general paediatricians – meeting seemed to be dominated by psychiatrists/psychologists (23)

The workshop was planned with definite areas for discussion and a very strong split between content and process. This defined structure led to cramping of ideas (27)

Maybe including some real SHOs just for the occasional reality check (14)

## Discussion

The workshop was valuable in contributing to the development of the Child in Mind Project training programme. The content and process of the programme were explored and several issues emerged that will need serious consideration by the Child in Mind project team. These include the strongly expressed need for a change in culture within the health care system that will embrace child-centred mental health care. The magnitude of change required is uncertain but may well be extensive given evidence that a study based in general practice in The Netherlands reported that the inclusion of the child in all phases of the consultation was "limited" with parents frequently speaking for the child, the child not questioning the parent, and the GP supporting this behaviour by minimal exploration of meta-communicative behaviours. The authors described this process resulting in a dyadic emphasis as being "institutionally co-constructed" [24].

Ways to change the health care culture in the United Kingdom were not explicitly identified. However, the project teams' desire to implement the training programme in a few centres that were already enthusiastic suggests that creating centres of best practice is inherent in their approach for change. This supports theoretical approaches for effective institutional change [25,26]. That is, implementation commences in sites receptive to change before introducing change on a wider scale having already demonstrated positive outcomes. One outcome of the workshop was the identification of individuals willing to trial the new programme with their trainees. These individuals work in centres with different structures and functions in the health care system so will prove valuable in evaluating how deliverable the programme is in different types of settings.

The inter-professional nature of the workshop was beneficial in exchanging views from different perspectives. This supports the findings of the few studies in medical curriculum development that reports this approach [27]. Most participants acknowledged the importance of continuing the consultation process although there was no attempt to agree on format. The importance of regular consultation with the principal users of the training programme – the senior house officers – will be essential to ensure that the programme is deliverable within the diverse settings in which they learn and work.

Although consultation with other stakeholders (children, adolescents and their families) was not identified by this group, it is important that they are also included in the development and evaluation of the training programme. Community participation – especially of key stakeholders, is often lacking in all phases of professional education (development, implementation and evaluation). In order that the training can best meet the needs of its intended targets their voices should be considered. The medical education literature strongly supports inclusion of patient voices in all aspects of curricula development [28-30].

The importance of training the trainers of the programme was identified as key to success of implementation. Although agreement was not sought, there was a powerful sentiment that trainers should be inter-professional. This notion may also address cultural barriers that relate to doctors' lack of understanding of other health care professional roles by exposing them to trainers who have mental health assessment and/or communication skills expertise. The nature of support provided to trainers may vary reflecting the diverse settings in which the training programme will eventually be implemented.

There appeared to be agreement that the workshop was not an appropriate forum for identifying the details of content and process of the training programme. Rather core issues were identified in psychosocial assessment, mental health and communication. Effective approaches to learning patient-centred communication skills are labour-intensive (videotaped interviews with feedback) [31,32] so maximising the benefits of such activities will be essential. The literature reports examples of communication skills programmes for trainee paediatricians [19,21] as well as other doctors and health care professionals who work with children [20,22,23,33] that address diverse issues. Common to many of these programmes is the use of simulated patients and parents incorporating critiquing of videotapes. This may provide valuable guidance in selecting educational methods that are effective and can be delivered in different settings. Ensuring that the training programme incorporates principles of work-based and other adult-learning approaches are essential [34-37].

The purpose of eliciting participants' reasons for attending and their expectations of the workshop is to help make sense of their satisfaction afterwards. Although the invitation outlined the purpose of the workshop, participants came with varied views that to some extent reflected their level of experience, their unique professional perspective and their interpretation of the information provided in the invitation. However, there was an overarching expectation that each would contribute to the development of a training programme. It is important to reflect on the reasons that only 28.6% of the participants reported that their expectations were completely met.

The suggestions given for improvements offer insight into why more participants did not meet their expectations. Restating the project team's aims at the commencement of the workshop may have been helpful. Although some participants felt able to express their views others were unable to do so because of the structure of sessions, the way in which they were facilitated and the settings in which the discussions took place. Providing a more open forum for discussion may have generated different ideas. The breadth and depth of the "culture change" some participants consider essential for implementation of the training programme is extensive and is likely to have influenced their judgement as to what could be realistically achieved both in the workshop and the training programme.

The physical limitations of the workshop impeded discussion in some groups.

Although group sizes were thought appropriate, providing spaces in which they could work will need to be considered in future workshops.

### Limitations of the evaluation

There are several limitations with this evaluation project some of which were beyond the control of the evaluator (DN).

• Higher response rates may have improved the quality of the evaluation. It is possible that respondents differed to non-respondents which may influence the results in someway although it is difficult speculate how.

• Scheduling the evaluation forms as part of the workshop may have increased response rates and may also help participants to focus on their expectations immediately before the meeting and then afterwards in considering what they achieved.

• The low response rate in relation to the final plenary session may be explained by the request to complete the forms immediately after the workshop. It is possible that some participants wanted more time to reflect on their experiences. It may have been more helpful to contact participants after the workshop.

• Further, the responses may not represent the diversity of opinions expressed during the workshop nor were the professional groups equally represented in the evaluation forms. For example, no nurses completed the pre workshop evaluation form. It is unclear why this was the case as all respondents were equally encouraged to complete the forms.

Future evaluations of workshops attended by disparate groups may consider:

• Scheduling the completion of evaluation forms into the workshop timetable

• Using identifiers to link pre and post workshop evaluation forms

• Following-up participants some time after the workshop to elicit their considered views

Despite these methodological weaknesses, the evaluation offers useful insights to the management of an inter-professional workshop for curricula development.

## Conclusions

The workshop provided the Child in Mind project team with valuable insight relevant to the development of a deliverable training programme in mental health and communication. This was an adequate forum in which the ideas and experiences of an interested inter-professional group could contribute although there were several ways in which this could have been improved. The diversity of the settings in which the programme will be delivered was highlighted as was the need for cultural change and support not only for trainees but the trainers themselves. Continued consultation with this inter-professional group together with broadening the consultation process to include other stakeholders may lead to the development of an effective training programme. Commencing the programme in sites with clinicians who are receptive to change of this nature is likely to influence its' success. Evaluation will continue to be essential to monitor the process. The enthusiasm of the participants needs to be harnessed to ensure that the long-term goals of the project team will be met.

## Competing interests

The author(s) declare that they have no competing interests.

## Authors' contributions

All authors contributed to each phase of the project although DN took a lead role in writing the paper. DN was responsible for the evaluation while ST and QS were instrumental in the development of the workshop.

## Pre-publication history

The pre-publication history for this paper can be accessed here:


